# Sexual Arousal Patterns of Identical Twins with Discordant Sexual Orientations

**DOI:** 10.1038/s41598-018-33188-2

**Published:** 2018-10-08

**Authors:** Tuesday M. Watts, Luke Holmes, Jamie Raines, Sheina Orbell, Gerulf Rieger

**Affiliations:** Department of Psychology, University of Essex, Colchester, UK

## Abstract

Genetically identical twins can differ in their self-reported sexual orientations. However, whether the twins’ subjective reports reflect valid differences in their sexual orientations is unknown. Measures of sexual orientation, which are free of the limitations of self-report, include genital arousal and pupil dilation while viewing sexual stimuli depicting men or women. We examined these responses in 6 male twin pairs and 9 female twin pairs who reported discordant sexual orientations. Across measures, heterosexual male twins responded more strongly to women than to men. Their homosexual co-twins showed an opposite pattern. Heterosexual female twins responded equally to both sexes, whereas their homosexual co-twins responded somewhat more to women than men. These differences within pairs were similar to differences between unrelated heterosexual and homosexual males and females. Our study provides physiological evidence confirming twins’ discordant sexual orientations, thereby supporting the importance of the non-shared environment for the development of sexual orientation and sexual arousal.

## Introduction

A recent review of twin studies indicates that although both genetic variations and the non-shared environment contribute to population-level differences in sexual orientation, the contribution of the non-shared environment is much stronger^1^. The non-shared environment is unique to each individual twin in an identical pair. Due to these unique influences, genetically identical twins can have discordant sexual orientations with one being heterosexual and the other homosexual.

Past research on identical twins with discordant sexual orientations has largely depended on their self-reports, which can be inaccurate^1^. Homosexual orientations, especially, may not be correctly disclosed. Some people are unaware that they are homosexual^2^. Others misrepresent their homosexual orientations because of stigma^3,4^. In some pairs of twins, both might identify as heterosexual, but one twin may not accurately disclose his/her homosexuality. In such cases, their true discordance would not be reflected in their identities. Or, both twins could be homosexual, but one twin may not disclose his or her same-sex preferences. In such cases, twins of a pair may be inaccurately identified as discordant. For these reasons, actual discordance could be different from what is implied by twins’ self-reports. As Bailey *et al*. stated in their review^1^: “Research supporting the validity of twin discordance—that twins who report different sexual orientations truly have them—is currently lacking, and would be most desirable for MZ [monozygotic] pairs.” The goal of the present research was to investigate the validity of the reported discordant sexual orientations of these twins.

Because self-report is not always accurate, some research has focused on assessing sexual orientation with other measures. Such measures include genital arousal to male and female sexual stimuli, assessed with penile gauges and vaginal plethysmographs. Another of these measures is the level of pupil dilation to sexual stimuli. In general, sex and sexual orientation differences in sexual response are comparable for these measures. Heterosexual men respond substantially more to females than males, and homosexual men respond substantially more to males than females^5–8^; thus, on average, genital arousal and pupil dilation patterns verify men’s reported sexual orientations.

Women of all sexual orientations respond, on average, with some level of arousal to erotic stimuli representing either sex. However, this effect is especially common in heterosexual women, whereas homosexual women respond somewhat more to the same sex than other sex. This pattern can be seen in women’s genital responses and pupillary responses. However, this pattern can vary for genital response, depending on stimulus type^9^. Moreover, for unknown reasons it is stronger if assessed with pupil dilation than genital response^7,8^. Pupil dilation is, on average, a valid indicator of sexual response^6,7,10,11^, but it cannot be ruled out that other cognitive or emotional effects distort its precision in this respect^12,13^. This could lead to somewhat different findings than achieved with measures of genital response. However, more important for the present research, is the overall pattern. The finding that heterosexual women are non-specific in their responses to males or females, whereas homosexual women respond more to their preferred sex, was confirmed with other measures, including reaction time, viewing time, neural activity, and thermography of genitalia^8,14–16^.

To the extent that self-reported differences in identical twins’ sexual orientations are accurate, their sexual responses will be similar to those of most heterosexual and homosexual individuals. Hence, in male pairs, we predicted that heterosexual twins would show substantially more sexual responses to the other sex, whereas the homosexual co-twins would show more arousal to the same sex. One previous study on the penile responses of one pair of identical twins confirmed this prediction^17^. In female pairs, we predicted that heterosexual twins would show similar sexual responses to both sexes, whereas their homosexual co-twins would show somewhat stronger sexual responses to the same sex than the other sex. Furthermore, if twins’ sexual responses reflect general sexual orientation differences, then their sexual responses should be similar in effect to the responses seen in unrelated heterosexual and homosexual individuals.

## Results

### Sexual Arousal of Male Twins

We predicted that heterosexual male twins show stronger sexual responses to the other sex, whereas their homosexual co-twins respond more strongly to the same sex. Initial one-sample t-tests indicated that heterosexual male twins showed more genital arousal to other-sex stimuli than to baseline: expressed as the average within-participant z-score (*Mz*), their arousal to the other sex was significantly larger than zero, *p* = 0.03, *Mz* = 1.68, 95% CI [0.23, 3.13]. These heterosexual twins did not show significantly more genital arousal to same-sex stimuli than baseline, *p* = 0.34, *Mz* = −0.33 [−1.18, 0.51]. Their homosexual co-twins had significantly more genital arousal to the same sex, *p* = 0.04, *Mz* = 1.57 [0.13, 3.00], but were not significantly more aroused to the other sex than baseline, *p* = 0.53, *Mz* = 0.08 [−0.23, 0.38].

**Figure 1:**
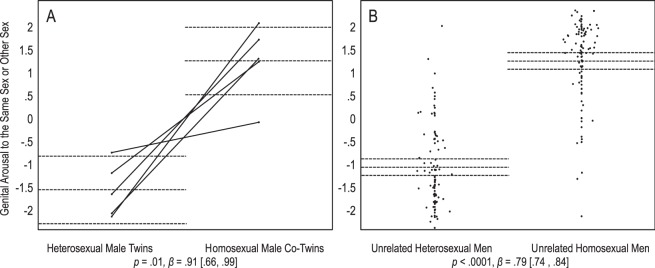
Men’s genital arousal to sexual stimuli. Panel A depicts 5 heterosexual twins and their 5 homosexual co-twins, and panel B depicts 94 unrelated heterosexual men and 97 unrelated homosexual men. On the Y axes, positive values indicate larger responses to the same sex than the other sex, and negative values indicate larger responses to the other sex than the same sex, *z*-scored within participants. Dots represent participants’ average scores. Full lines connect twins of pair. Dotted lines represent group means with 95% confidence intervals. Statistics are main effects of sexual orientation on response to the same or other sex. For twins, dependency of data was accounted for by including pairs as a random effect.

We then conducted a mixed-factorial regression analysis. The dependent variable was the contrast of genital arousal to the same sex or other sex. The independent variable was sexual orientation as a fixed effect. Twin pairs were included as a random effect to account for dependency of the data within pairs. Heterosexual male twins had more genital arousal to the other sex, and their homosexual co-twins showed more arousal to the same sex. This difference in sexual orientation was significant, *p* = 0.01, *β* = 0.91 [0.66, 0.99] (Fig. 1A).

**Figure 2:**
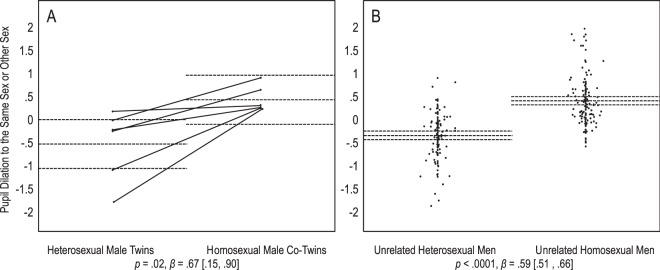
Men’s pupil dilation to sexual stimuli. Panel A depicts 6 heterosexual twins and their 6 homosexual co-twins, and panel B depicts 123 unrelated heterosexual men and 136 unrelated homosexual men. On the Y axes, positive values indicate larger responses to the same sex than the other sex, and negative values indicate larger responses to the other sex than the same sex, *z*-scored within participants. Dots represent participants’ average scores. Full lines connect twins of pair. Dotted lines represent group means with 95% confidence intervals. Statistics are main effects of sexual orientation on response to the same or other sex. For twins, dependency of data was accounted for by including pairs as a random effect.

In their pupil dilation, heterosexual male twins responded significantly to both sexes compared with baseline, but somewhat more to the other sex, *p* = 0.03, *Mz* = 1.12 [0.14, 2.09], than the same sex, *p* = 0.02, *Mz* = 0.72 [0.13, 1.31]. Their homosexual co-twins also responded significantly to both sexes, but somewhat more so to the same sex, *p* = 0.03, *Mz* = 1.17 [0.15, 2.18], than the other sex, *p* = 0.04, *Mz* = 0.92 [0.00, 1.81]. Next, we regressed the contrast of pupil dilation to the same sex or other sex onto sexual orientation. Heterosexual male twins dilated more to the other sex, and their homosexual co-twins dilated more to the same sex. This sexual orientation difference was significant, *p* = 0.02, *β* = 0.67 [0.15, 0.90] (Fig. 2A).

### Sexual Arousal of Female Twins

We predicted that heterosexual female twins show similar sexual responses to both sexes, but their homosexual co-twins respond more strongly to the same sex. Heterosexual female twins had significant genital arousal to the same sex and the other sex, each compared with baseline, *p* = 0.0002, *Mz* = 2.02 [1.51, 2.53], and *p* = 0.0001, *Mz* = 1.95 [1.61, 2.29], respectively. Their homosexual female co-twins also showed genital arousal to the same sex and other sex, each compared with baseline, although somewhat less so for the latter, *p* = 0.001, *Mz* = 1.92 [1.17, 2.67], and *p* = 0.01, *Mz* = 1.18 [0.40, 1.97].

**Figure 3:**
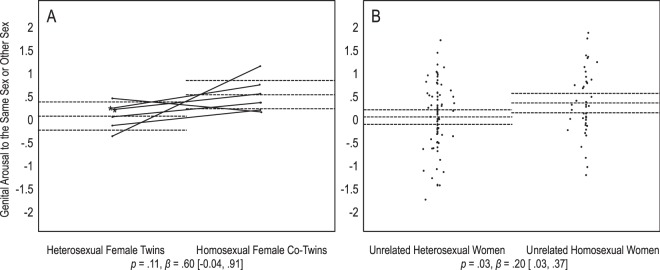
Women’s genital arousal to sexual stimuli. Panel A depicts 6 heterosexual twins and their 6 homosexual co-twins, and panel B depicts 77 unrelated heterosexual women and 44 unrelated homosexual women. On the Y axes, positive values indicate larger responses to the same sex than the other sex, and negative values indicate larger responses to the other sex than the same sex, *z*-scored within participants. Dots represent participants’ average scores. Asterisks represent imputed average scores of two heterosexual female twins. Full lines connect twins of pair. Dotted lines represent group means with 95% confidence intervals. Statistics are main effects of sexual orientation on response to the same or other sex. For twins, dependency of data was accounted for by including pairs as a random effect.

We then regressed female twins’ genital arousal to the same sex or other sex onto their sexual orientation. Twin pairs were a random effect. Heterosexual female twins responded about equally to both sexes (the 95% confidence intervals of their average contrast score included the value of 0). In comparison, their homosexual co-twins showed somewhat more arousal to the same sex. Although in the predicted direction, this difference in sexual orientation was not significant, *p* = 0.11, *β* = 0.60 [−0.04, 0.91] (Fig. 3A).

**Figure 4:**
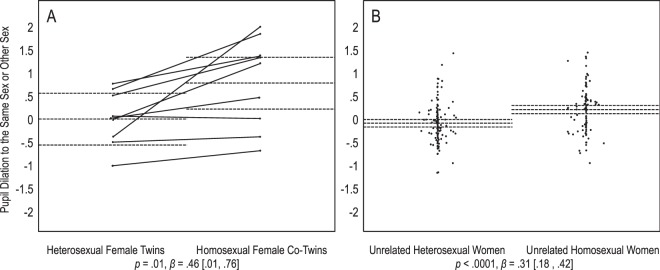
Women’s pupil dilation to sexual stimuli. Panel A depicts 9 heterosexual twins and their 9 homosexual co-twins, and panel B depicts 124 unrelated heterosexual women and 103 unrelated homosexual women. On the Y axes, positive values indicate larger responses to the same sex than the other sex, and negative values indicate larger responses to the other sex than the same sex, *z*-scored within participants. Dots represent participants’ average scores. Full lines connect twins of pair. Dotted lines represent group means with 95% confidence intervals. Statistics are main effects of sexual orientation on response to the same or other sex. For twins, dependency of data was accounted for by including pairs as a random effect.

In their pupil dilation patterns, heterosexual female twins responded significantly to both the same sex and other sex, each compared with baseline, *p* = 0.02, *Mz* = 0.63 [0.12, 1.23], and *p* = 0.02, *Mz* = 0.69 [0.11, 1.26], respectively. Their homosexual female co-twins also responded to both sexes, but had stronger responses to the same sex than the other sex, each compared with baseline, *p* = 0.0008, *Mz* = 1.14 [0.63, 1.64], and *p* = 0.01, *Mz* = 0.65 [0.17, 1.23], respectively. Finally, we regressed their pupil dilation to the same sex or other sex onto their sexual orientation. Heterosexual female twins showed equal pupil dilation to both sexes, whereas their homosexual female twins responded more to the same sex. This sexual orientation difference was significant, *p* = 0.01, *β* = 0.46 [0.01, 0.76] (Fig. 4A).

### A Comparison of Twins with Unrelated Participants

We predicted that the difference in the twins’ sexual responses is comparable to the difference between unrelated heterosexual and homosexual individuals. Results were consistent with this prediction. In most cases, the 95% confidence intervals of heterosexual and homosexual twins’ average responses included the means of the corresponding heterosexual and homosexual unrelated groups (Figs 1–4). A set of regression analyses indicated that for male and female genital arousal, and male pupil dilation, twins and unrelated individuals did not significantly differ in the effect of sexual orientation on sexual response (0.39 < *ps* < 0.70, 0.03 < *βs* < 0.04, −0.20 < 95% CIs < 0.21). However, homosexual female twins dilated more strongly to the same sex than the other sex, as compared with unrelated homosexual women. The difference between twins and unrelated women in the effect of sexual orientation was significant, *p* = 0.04, *β* = 0.12 [0.01, 0.25]. We further note that unlike heterosexual female twins, unrelated heterosexual women dilated significantly more to the other sex than the same sex, *p* = 0.89, *Mz* = −0.04 [−0.48, 0.39], and *p* = 0.0008, *Mz* = −0.13 [−0.21, −0.06], respectively (Fig. 4A,B).

## Discussion

The present study suggests that identical twins with discordant self-reported sexual orientations differ in their patterns of physiological sexual arousal in a manner similar to unrelated heterosexual and homosexual individuals. These findings support the validity of identical twins’ self-reported discordance in sexual orientation.

Approximately 3% of the population identifies as gay, lesbian, or bisexual^18^, and 0.4% of the population are identical twins^19^. Thus, a simple estimate is that 3% out of 0.4% = 0.012% of the population are identical twins who are homosexual or bisexual. The percentage of those from discordant pairs is even smaller, and we aimed to have both twins of a pair in our lab. Given these prerequisites, it is noteworthy that we were able to collect data from 30 individual twins with discordant sexual orientations. Still, some true differences might not have been detected due to a lack of statistical power. Furthermore, we cannot rule out that there are other pairs of twins than those recruited that are truly discordant but did not identify as such (and thus, did not participate), or twins who report that they are discordant even though they are concordant (i.e. concordant homosexual) and we were not able to recruit them.

Factors other than genetics must account for differences in identical twins’ sexual orientation and arousal. In theory, a combination of epigenetic and prenatal hormonal influences could affect the formation of human sexual orientation^20–24^. In addition, there is increasing evidence that mothers can have immunological reactions that lead to their sons’ homosexuality^25–27^. Any of these influences could lead to the twins’ discordances, but as of today, there is no clear evidence that these factors affect identical twins’ sexual orientations differently.

Findings were not entirely alike for the two measures used in this study. For example, male twins did not show statistically significant genital arousal to stimuli showing their non-preferred sex, but they did show significant pupil dilation to those stimuli. Although there is much evidence for the validity of genital arousal as an indicator of sexual interest (especially for men)^28–30^, the application of pupil dilation to this domain is more recent. Even though there is evidence for pupil dilations’ general validity as measure of sexual response^6,10^, there are still potential, unknown factors that might affect its precision. For instance, pupil dilation does not only reflect sexual interest, but also emotions, cognitions, or reactions to any other stimuli that cause interest^12,13^. It is possible that male twins’ increased dilation to their non-preferred sex were due to such other influences. Because the assessment of pupil dilation is becoming more refined^10^, the future will tell to what degree it can ever be a measure that fully reflects sexual responses. However, even if the measure of pupil dilation turns out to be a less precise measure of sexual arousal, the twins’ genital responses still indicated that they differed in predicted ways.

Our study verified the discordant sexual orientations of genetically identical twins by comparing their self-reports to their physiological sexual responses. Thus, consistent with the overall conclusion from other research^1,20^, environmental factors unique to an individual can have a substantial influence on sexual preferences and physiological sexual arousal.

## Method

The University of Essex’s Ethics Committee approved this study (“GR1303”). The research followed the approved procedures and all participants gave informed consent.

### Participants

#### Twins

We advertised for identical twins with discordant sexual orientations via newsletters at UK universities, social media sites, online news sites for gay men and lesbians, and at three gay Pride festivals. Respondents included 6 male twin pairs and 9 female twin pairs reporting discordant sexual orientations, for a total of 30 individual twins. On a questionnaire, twins chose categories to self-identify as “straight,” “bisexual,” “gay” or “lesbian.” A final category was “other - please describe.” No twin indicated an identity in the “other” category.

The number of female twins who identified as bisexual (2) was low relative to the number of straight women and lesbians (9 and 7). Furthermore, these bisexual females reported a stronger preference for women than for men. Specifically, on a Kinsey scale, they scored 4 and 5 (with 6 meaning exclusive homosexual orientation). Including or excluding the two bisexual females did not substantially affect results, and they were included in all analyses. For the sake of simplicity, we grouped bisexual women with lesbians into a “homosexual” group. Likewise, gay male twins were labelled “homosexual;” straight twins were labelled “heterosexual.” For the 6 male pairs, the mean age (SD) was 25.00 (9.19) and 100% were White. The mean age of the 9 female pairs was 26.22 (4.52) and 89% were White and 11% were Black.

For males, predicted sexual orientation effects (standardized regression coefficients *βs*) on genital arousal or pupil dilation to male or female stimuli fall between 0.70 and 0.90^6^. Thus, significant power of 0.80 can be achieved with a minimum of 5 heterosexual and 5 homosexual males. The sample of male twins met this minimum. For females, predicted sexual orientation effects fall between 0.20 and 0.30. Thus, significant power of 0.80 may only be achieved with a larger sample (a minimum of 80 women) than the recruited 9 heterosexual twins and 9 co-twins. For the weak effect, sexual orientation differences in female sexual arousal are often not significant, and a focus may be given on the expected difference in effect rather than on level of significance. Furthermore, identical twins with discordant sexual orientations, willing and able to come to a lab, are difficult to find. For this reason it is scientifically informative to examine their physiological sexual arousal patterns, even if their numbers are small.

Twins’ monozygosity was confirmed via DNA analyses from saliva samples, conducted by Genetrack Biolabs UK. In addition, five questions about physical and visual similarity were administered^31^. A sample question is “During childhood, could you ever have fooled friends by pretending to be your twin?” Items were assessed on scales ranging from 1 to 3, with lower scores reflecting higher similarity. For all twins, their average scores were approximately 1, suggesting monozygosity. Similar questions about zygosity are usually 95% accurate or higher, based on comparisons with blood group or DNA analyses^32^.

#### Unrelated participants

Unrelated participants took part in previously conducted studies and included 94 heterosexual men, 97 homosexual men, 77 heterosexual women, and 44 homosexual women with genital arousal data, and 123 heterosexual men, 136 homosexual men, 124 heterosexual women, and 103 homosexual women with pupil data^5,6,8,33,34^. Their mean age (SD) was 24.89 (6.83). The majority was White (69%), followed by Black (9%), Hispanic (7%), South Asian (4%), East Asian (2%), and other ethnicities.

### Material and Measures

#### Stimuli

Six 3-minute videos, three of which showed males and three showed females, were used as sexual stimuli. Videos had similar content (a naked person in a bedroom) and depicted either a male model or female model masturbating. In a pilot study, these videos were selected from a pool of 200 videos drawn from the Internet. Heterosexual and homosexual men and women rated these models on their sexual appeal, and the three highest-rated male and female models across all rater groups were used for the experiment.

Six 2-minute videos were taken from a nature documentary for assessing baseline genital responses. Their engaging but nonsexual content facilitated participants’ return to an unaroused baseline. However, because their engaging content could elicit pupillary responses for reasons other than sexual interest, for pupil data two 2-minute animations of clouds were used for assessing baseline. All videos were of similar luminance; furthermore, luminance was set to equal upper and lower thresholds across stimuli by using the programs MPEG Streamclip and Final Cut Pro. Videos had a resolution of 768 by 536 pixels, and were presented full screen.

#### Genital data

A BIOPAC MP150 data acquisition unit and the program AcqKnowledge recorded genital responses every 5 milliseconds. An indium/gallium strain gauge measured changes in penile circumference. The signal was sampled at 200 Hz, low-pass filtered to 10 Hz and digitized with 16 bits resolution. Gauges were calibrated over six 5-mm steps before sessions and signals were transformed into millimeters of circumference.

Women’s genital arousal was assessed via change in vaginal pulse amplitude (VPA), using a vaginal photoplethysmograph. The VPA signal was sampled at 200 Hz, and high-pass filtered at 0.5 Hz with 16 bits resolution. VPA was measured as peak-to-trough amplitude for each pulse. VPA signals exhibit both convergent and discriminant validity of female sexual response^30^.

Of the 6 male twin pairs, 5 pairs agreed to have their genital arousal measured. Hence, for this measure, 5 heterosexual male twins were compared with 5 homosexual co-twins. Of the 9 females pairs, individuals from 6 female pairs consented to have their genitals measured. For 2 out of these 6 pairs, only one twin provided genital data. Hence, for this measure, the original numbers were 4 heterosexual female twins and 6 homosexual co-twins. In order to create a complete data set across these 6 female pairs, missing data of these two heterosexual female twins were imputed, based on the covariance of female twins’ sexual orientation with their genital arousal to the same sex and other sex^35^. Findings were comparable in effect and level of significance, with or without these imputations. Results with imputed data are reported.

#### Pupil data

An infrared gaze tracker from SR Research recorded pupil data every millisecond with a 35 mm lens focused on participants’ preferred eye. The program EyeLink computed pupil area as the number of the tracker’s camera pixels occluded by the infrared light reflected by the pupil. If pupils dilated while viewing stimuli, more pixels were occluded. Both twins in all 6 male pairs and all 9 female pairs had their pupils measured.

### Procedure

Twins provided written informed consent and were seated in a dimly lit room facing a screen with resolution of 1024 by 768 pixels. Their heads were approximately 500 mm away from the gaze tracker’s lens. For calibration of their pupil data, twins fixated and re-fixated their gaze on 9 points that defined the outline of the screen. Next, in privacy, males placed the gauges midway around their penises and females inserted the photoplethysmograph. Eye movements were then remotely recalibrated. Twins were instructed to watch all videos carefully. First, they watched an animation of clouds followed, in random order, by presentations of same-sex and other-sex sexual stimuli alternating with nature scenes. The final video was the second animation of clouds. After the session participants were paid. The procedure took 60 minutes. Procedures for unrelated participants were almost identical to those described for twins^5,6,8,33,34^.

For each participant, both genital data and pupil data were averaged within each stimulus. We then computed z-scores of these averages within participants. For genital data, standardized responses to the 10 seconds preceding a sexual stimulus (at the end of a neutral stimulus and at which time they had returned to baseline) were subtracted from the standardized response to this stimulus. For pupil data, standardized responses to neutral (the animated clouds) were subtracted from standardized responses to all other stimuli. We next computed, for each participant, average values across stimuli of the same type, reflecting his or her overall genital response and pupil response, respectively, to same-sex stimuli and other-sex stimuli. Finally, for each participant, we computed contrast scores representing responses to the same sex or the other sex. At request, data can be made available to readers.
